# Using Large Language Models to Summarize Evidence in Biomedical Articles: Exploratory Comparison Between AI- and Human-Annotated Bibliographies

**DOI:** 10.2196/69707

**Published:** 2026-02-12

**Authors:** Michelle Colder Carras, Riaz Qureshi, Kevin Naaman, Faisal Aldayel, Mayank Date, Dahlia AlJuboori, Johannes Thrul

**Affiliations:** 1 Department of International Health, Johns Hopkins Bloomberg School of Public Health, 615 N Wolfe St, Baltimore, MD, 21205, United States, 1 410-955-3934; 2 Department of Ophthalmology, School of Medicine, University of Colorado Anschutz Medical Campus, Aurora, CO, United States; 3 Department of Mental Health, Johns Hopkins Bloomberg School of Public Health, , Baltimore, MD, United States

**Keywords:** annotated bibliography, artificial intelligence, ChatGPT, evidence synthesis, information management, large language model

## Abstract

**Background:**

Annotated bibliographies summarize literature, but training, experience, and time are needed to create concise yet accurate annotations. Summaries generated by artificial intelligence (AI) can save human resources, but AI-generated content can also contain serious errors.

**Objective:**

To determine the feasibility of using AI as an alternative to human annotators, we explored whether ChatGPT can generate annotations with characteristics that are comparable to those written by humans.

**Methods:**

We had 2 humans and 3 versions of ChatGPT (3.5, 4, and 5) independently write annotations on the same set of 15 publications. We collected data on word count and Flesch Reading Ease (FRE). In this study, 2 assessors who were masked to the source of the annotations independently evaluated (1) capture of main points, (2) presence of errors, and (3) whether the annotation included a discussion of both the quality and context of the article within the broader literature. We evaluated agreement and disagreement between the assessors and used descriptive statistics and assessor-stratified binary and cumulative mixed-effects logit models to compare annotations written by ChatGPT and humans.

**Results:**

On average, humans wrote shorter annotations (mean 90.20, SD 36.8 words) than ChatGPT (mean 113, SD 16 words) which were easier to interpret (human FRE score, mean 15.3, SD 12.4; ChatGPT FRE score, mean 5.76, SD 7.32). Our assessments of agreement and disagreement revealed that one assessor was consistently stricter than the other. However, assessor-stratified models of main points, errors, and quality/context showed similar qualitative conclusions. There was no statistically significant difference in the odds of presenting a better summary of main points between ChatGPT- and human-generated annotations for either assessor (Assessor 1: OR 0.96, 95% CI 0.12‐7.71; Assessor 2: OR 1.64, 95% CI 0.67‐4.06). However, both assessors observed that human annotations had lower odds of having one or more types of errors compared to ChatGPT (Assessor 1: OR 0.31, 95% CI 0.09‐1.02; Assessor 2: OR 0.10, 95% CI 0.03‐0.33). On the other hand, human annotations also had lower odds of summarizing the paper’s quality and context when compared to ChatGPT (Assessor 1: OR 0.11, 95% CI 0.03‐0.33; Assessor 2: OR 0.03, 95% CI 0.01‐0.10). That said, ChatGPT’s summaries of quality and context were sometimes inaccurate.

**Conclusions:**

Rapidly learning a body of scientific literature is a vital yet daunting task that may be made more efficient by AI tools. In our study, ChatGPT quickly generated concise summaries of academic literature and also provided quality and context more consistently than humans. However, ChatGPT’s discussion of the quality and context was not always accurate, and ChatGPT annotations included more errors. Annotated bibliographies that are AI-generated and carefully verified by humans may thus be an efficient way to provide a rapid overview of literature. More research is needed to determine the extent that prompt engineering can reduce errors and improve chatbot performance.

## Introduction

The fields of medicine and public health are dynamic and constantly evolving, with a continuous influx of new research articles and evidence. Staying up to date with the latest literature poses a significant challenge for researchers and practitioners alike [1], and it is difficult to familiarize oneself with the literature when diving into a new subject area [2]. Evidence summaries such as meticulously curated reviews and guidelines are lengthy and often complex, and their number has exponentially increased over the past decade [3,4]. In the past, individuals (oftentimes students) may have resorted to sources such as blogs or Wikipedia to understand the basics of a topic, ideally before diving into more reputable peer-reviewed publications. However, this approach has been superseded by use of artificial intelligence (AI) large language models (LLMs) such as ChatGPT, which provide summaries based on accessible scientific literature but often have questionable accuracy and frank errors [5,6].

As a research tool, annotated bibliographies may be a valuable remedy for efficient and insightful literature exploration. These condensed summaries highlight the main points, findings, and scientific validity of the respective articles, while also providing context by relating them to existing knowledge within the field [7].

Recent studies on the use of LLMs show mixed evidence for these as evidence synthesis methodologies. While newer models may provide good summaries, it is unclear whether they have improved in their ability to effectively summarize and analyze scientific materials [5,6,8,9]. AI chatbots and LLMs may have limitations in capturing nuances and answering specific requests accurately, in addition to their known challenges of hallucinating information and citations [5,6,9]. These limitations may stem from an inability to discern the main points of articles (for example, distinguishing factual statements in findings from background material) and may result in output that diverges from the intended context [10]. On the other hand, developing the skill to write succinct and insightful annotations requires time and practice, as well as a solid background knowledge of the subject matter [11]. LLMs with specialized training datasets or access to current bibliographic databases have the ability to provide output based on a breadth and depth of knowledge that other LLMs likely do not, making them an even more promising technology [12]. Keeping abreast of recent advancements and understanding them in the context of existing knowledge can be a daunting task, even for experienced professionals in the field.

Given the varying opinions about the utility of LLMs in generating summaries and citing them in context, our objective was to explore the quality and comparability of annotated bibliographies produced by authors of varying professions and levels of training with outputs generated by a widely available LLM-powered chatbot, ChatGPT [13]. By describing the strengths and weaknesses of both human-authored and ChatGPT-generated annotated bibliographies, we aim to explore the potential utility of ChatGPT in the context of literature exploration and synthesis in medicine and public health.

## Methods

### Overview

We conducted an exploratory study to describe the similarities and differences in characteristics (word count and readability) and subjective assessments from 2 assessors (errors, inclusion of main points, and presence or absence of quality assessment and contextualization) of annotated bibliographies that were created by 2 researchers (JT and MD) and 3 versions of ChatGPT (ChatGPT, models GPT-3.5, 4, and 5, OpenAI) [13]. Our data, statistical code, and research materials are available on the Open Science Framework (OSF) [14] and we provide links and descriptions to each OSF file (Table S1 in the Multimedia Appendix 1).

### Ethical Considerations

Ethics approval was not required because this study involved secondary analysis of published data and did not include human participants.

### Annotation Creation

#### Article Selection

We searched Web of Science for publications in life and biomedical sciences published from May 1, 2013 to May 1, 2023. From this search, we sampled 15 publications [15-29] that were the most highly cited, open access, and had 15 or fewer pages of main text. Our full article selection procedure, including search strategy and sampling methods, is described on OSF (Table S1 in the Multimedia Appendix 1).

#### Human Annotators and Chatbot Prompters

Two human annotators (JT, a professor in public health, and MD, a dentist and recent public health master’s degree graduate) and 3 chatbot prompters (MD, the same master’s degree graduate; FA, a graduate student; and RQ, an epidemiologist specializing in evidence synthesis) from our research team created annotated bibliographies using the same set of 15 publications. Each chatbot prompter was restricted to using one version of ChatGPT. MD was a human annotator and a chatbot prompter. To avoid MD being influenced by annotations written by ChatGPT, MD read and annotated all 15 annotations before beginning his role as a chatbot prompter.

The human annotators and chatbot prompters completed their annotations in August 2023. To update our assessment during the peer review process in September 2025, RQ used ChatGPT-5 to create another set of annotations using the latest version of ChatGPT.

#### Directions Given to Human Annotators and Chatbot Prompters

The 2 human annotators (JT and MD) and 3 chatbot prompters (MD, FA, and RQ) received instructions from members of our research team on how to write an annotated bibliography. RQ instructed the human annotators to review 1 video and 2 webpages that described how to write an annotated bibliography (Box S1 in the Multimedia Appendix 1).

MCC emailed instructions to the chatbot prompters (Box S2 in the Multimedia Appendix 1) on how to create ChatGPT-generated annotations, including uploading the text of each article into ChatGPT using the web-based ChatGPT Splitter [30] and using a naturalistic approach with few limits to prompt generation of summaries. As chatbot annotations were completed in August 2023 prior to ChatGPT being able to process PDF files directly, a text file was generated from each PDF. An initial pilot test demonstrated that ChatGPT produced long summaries, so instructions were updated to restrict responses to 90-150 words to be more consistent with human annotations, providing better masking. ChatGPT-5 was restricted to 3-4 sentences. We altered the prompt for ChatGPT-5 to sentences rather than word count to see what kind of effect this would have on the resulting word counts.

### Project Management and Workflow

For the human annotations, the 15 publications were uploaded to PICO Portal [31], where the human annotators read each publication, wrote their annotations, and documented how long it took to complete each annotation in minutes.

Chatbot prompters who reported that the prompt did not generate a summary were instructed to approach the situation however they wanted to (eg, using additional prompts or the “Regenerate response” button) until a summary was created. Researchers who generated responses through AI designated a final annotation that was then scrubbed (see example below) and used in analysis. All annotations were created in single ChatGPT sessions without clearing ChatGPT’s memory between citations. All ChatGPT conversations are available on OSF (links can be found in Table S1 in the Multimedia Appendix 1).

The chatbot annotations were scrubbed to remove formatting anomalies and recurrent patterns which may have unmasked ChatGPT-generated annotations. For example, ChatGPT often added titles or citation information to responses. An example of scrubbing an original annotation is shown below:

Selected Text From Original Response (ChatGPT-3.5)

Title: Theta Band Activities and Cognitive Control: A Mechanistic Perspective. This article provides an insightful overview of recent advancements in cognitive neuroscience.

Scrubbed Version Used in Analysis

This article provides an insightful overview of recent advancements in cognitive neuroscience.

Additional examples of scrubbing original annotations are presented in Table S2 in the Multimedia Appendix 1.

We did not scrub body text of the annotations themselves, even if they contained clear errors. For example, the article by Cavanagh and Frank [17] is a narrative review that discusses the role of frontal theta band activities in the medial prefrontal cortex as they relate to cognitive control mechanisms. Yet, ChatGPT-3.5 incorrectly stated the publication focused on the “mid-frontal cortex” while ChatGPT-4 said the research “employ[ed] a variety of experimental methods.” Additional examples of errors can be found in Table S3 in the Multimedia Appendix 1.

After scrubbing annotations created by ChatGPT, we combined all annotations for each article (n=75 annotations in total) into a Microsoft Excel file and numbered them individually. We randomly ordered all annotations using random.org and masked the source of the annotations by removing the column which identified the versions of ChatGPT or the human annotator that produced each annotation. We used this file to collect data on the following outcome measures.

### Outcome Measures and Definitions

For each annotation, we assessed (1) word count, (2) Flesch Reading Ease (FRE), (3) capture of main points, (4) presence and severity of errors, (5) presence of a quality assessment and contextualization of the publication’s results, and (6) we guessed whether the annotation was generated by AI or a human. We entered each annotation into a web-based calculator to measure word count and FRE. FRE is a reliable test with no grade-level ceiling that measures how difficult it is for a person to read and understand written material [32]. Scores range from 0 to 100, where higher scores indicate easier to understand material (ie, grade or middle-school students), and lower scores indicate harder to understand material (ie, college graduates or professionals) [33].

Two epidemiologists (RQ and KN) specializing in evidence synthesis methods independently evaluated the 3 remaining outcome measures (eg, main points, errors, and presence or absence of quality assessment and contextualization). First, they rated main points on an ordinal scale from 1 to 7, where a score of 1 indicated the annotation included little relevant information, a score of 4 indicated the annotation focused on minor rather than major points of the publication, and a score of 7 indicated the annotation included all relevant information about the publication’s main purpose. Second, they measured the extent of errors in each annotation using four ordinal categories: no errors, one minor error, one major error, or multiple errors. Errors were measured by ensuring each claim in the annotation appeared in the main text of the publication. Third, they assessed whether each annotation discussed the quality of the study (ie, risk of bias, methodology) and contextualized the study’s results in comparison to the body of literature. They assigned a value of “yes” if *both* quality and context were present, regardless of whether quality and context were technically incorrect or irrelevant to the publication, and a value of “no” if *either* quality or context were missing from the annotation. If quality and context were *both* present but incorrect, this was still counted as “yes” but also counted towards the assessment of errors in the annotation.

### Analysis

We analyzed data using R software (version 4.5.1; R Foundation for Statistical Computing) [34], and the *tidyverse* [35], *psych* [36], *ordinal* [37], and *lme4* [38] packages.

#### Word Count and Flesch Reading Ease

We calculated measures of central tendency and spread for word count and FRE for all 5 annotators. We first aggregated analysis of central tendency and spread by the 2 human annotators vs the 3 versions of ChatGPT, then split into individual annotators (2 humans and 3 ChatGPT versions). Due to the exploratory nature of our study, we did not conduct inferential tests on differences in these measures.

#### Assessing Interrater Agreement and Disagreement

To better address aspects of subjectivity in our expert ratings of annotations, we evaluated interrater agreement between the 2 assessors using Cohen kappa for the quality and context binary rating and weighted Cohen kappa for the ordinal variables (main points and errors). Following the recommendations by Agresti [39], we also investigated patterns for systematic disagreement by applying the McNemar test for the quality and context rating (without continuity correction) and ordinal quasi-symmetry models for main points and errors. See Text S1 in the Multimedia Appendix 1 for additional details.

#### Main Points, Errors, Quality and Context, and Guesses

We first calculated frequencies and percentages of each response option for both assessors’ evaluations of main points, errors, quality and context, and if they correctly guessed whether annotations were written by a human or ChatGPT. Because RQ was the chatbot prompter for ChatGPT-5, he did not guess the source of the 15 annotations generated by ChatGPT-5. We then fit assessor-stratified binary and cumulative mixed-effects logit models with a binary predictor for source of annotation (human vs ChatGPT). We always set ChatGPT as the reference group. Additional details on our modeling strategies, including our assessment of the proportional odds assumption and whether we needed a random intercept for publication or annotators are reported in Text S1 in the Multimedia Appendix 1.

Statistical significance was defined as *P*<.05; however, because the goal of our analysis was exploratory, we reported results with a focus on describing our observed data rather than intending to draw inferences to a population of studies beyond the scope of this paper.

## Results

### Overview

Each annotator and chatbot prompter produced one annotation for each of the 15 publications, which resulted in 75 annotations. On average, it took the professor 13.7 (SD 3.4) minutes and the master’s degree graduate 21.1 (SD 15.7) minutes to write 1 annotation. Chatbot prompters observed that ChatGPT generated summaries within seconds per publication, but without timestamps being available, we did not measure the exact time.

### Word Count and Flesch Reading Ease

When aggregated by chatbot vs human, ChatGPT annotations were longer and had less variation in word count (mean 113, SD 16) than human annotators (mean 90.2, SD 36.8). ChatGPT annotations had lower average FRE scores (mean 5.76, SD 7.32) than the human annotators (mean 15.3, SD 12.4), although both chatbots and human annotations fell in the “very difficult” range [40].

Individually, the annotations generated by ChatGPT-3.5 and 4 always stayed within the lower and upper bounds of their restricted word counts of 90-150 words. ChatGPT-5’s word count distribution was almost identical to ChatGPT-3.5’s word count, even though ChatGPT-5 had a 3‐4 sentence limit rather than a word count limit (Table 1).

**Table 1.** Word count and Flesch Reading Ease score for 15 annotations.

	Mean (SD)	Median (minimum-maximum)
Word count		
Overall ChatGPT^a^	113 (16)	114 (86-148)
GPT-3.5	125 (113)	124 (105-148)
GPT-4	97.8 (10.2)	96 (86-119)
GPT-5	117 (12.2)	120 (102-148)
Overall human^a^	90.2 (36.8)	79.5 (41-170)
MD (MPH graduate)	68.8 (25.1)	63 (41-143)
JT (professor)	111.6 (34.6)	120 (61-170)
Flesch Reading Ease score^b^		
Overall ChatGPT^a^	5.76 (7.32)	2.6 (0-22.4)
GPT-3.5	6.95 (7.43)	4.9 (0-20.7)
GPT-4	9.11 (8.56)	10 (0-22.4)
GPT-5	1.22 (2.07)	0 (0-6.4)
Overall human^a^	15.3 (12.4)	14.6 (0-37)
MD (MPH graduate)	15.9 (13.5)	15 (0-37)
JT (professor)	14.6 (11.7)	14.2 (0-34)

a Aggregated results by annotator (ChatGPT or human)

b Flesch Reading Ease score potential range is from 0 to 100, with 0 indicating low readability (scientific level, eg journal articles) and 100 indicating high readability (very easy, eg, comic books) [40].

### Assessing Interrater Agreement and Disagreement

Detailed results for assessing agreement and disagreement are reported in Text S1, Tables S4-S8, and Figures S1-S3 of the Multimedia Appendix 1. Briefly, Cohen κ showed that assessors had fair to moderate levels of agreement for their assessment of main points (κ=0.32, 95% CI 0.11‐0.52), errors (κ=0.43, 95% CI 0.23‐0.63), and quality and context (κ=0.46, 95% CI 0.26‐0.67). As for disagreement, ordinal quasi-symmetry models revealed systematic disagreement where one assessor was stricter than the other. The stricter assessor had lower odds of assigning higher categories of main points (OR 0.49, 95% CI 0.30‐0.71) and higher odds of assigning higher (ie, worse) categories of errors (OR 3.05, 95% CI 1.69‐7.56). For quality and context, there was no evidence of systematic disagreement (McNemar χ^2^=0.2, *P*=.655). Despite our assessments of the raters’ levels of agreement or disagreement, the assessor-stratified models in Table 2 showed that both assessors’ ratings resulted in similar qualitative conclusions about main points, errors, and quality and context.

**Table 2.** Assessors’ ratings of 45 ChatGPT- and 30 human-annotated bibliographies^a^.

	Assessor 1			Assessor 2		
Indicator	GPT, *f* (%)^b^	Human, *f* (%)	Statistical tests, OR (95% CI)^c^	GPT, *f* (%)	Human, *f* (%)	Statistical tests, OR (95% CI)^c^
Main points			0.96 (0.12‐7.71)			1.64 (0.67‐4.06)
1	1 (2)	0 (0)		5 (11)	1 (3)	
2	0 (0)	0 (0)		4 (9)	0 (0)	
3	4 (9)	1 (3)		1 (2)	1 (3)	
4	5 (11)	4 (13)		6 (13)	6 (20)	
5	5 (11)	7 (23)		10 (22)	8 (27)	
6	16 (36)	9 (30)		16(36)	13 (43)	
7	14 (31)	9 (30)		3 (7)	1 (3)	
Errors			0.31 (0.09‐1.02)			0.10 (0.03‐0.33)
None	29 (64)	25 (83)		16 (36)	22 (73)	
1 Minor	7 (16)	3 (10)		4 (9)	5 (17)	
1 Major	1 (2)	1 (3)		5 (11)	1 (3)	
Multiple	8 (18)	1 (3)		20 (44)	2 (7)	
Quality and context			0.11 (0.03‐0.33)			0.03 (0.01‐0.10)
Absent	12 (27)	22 (73)		9 (20)	27 (90)	
Present	33 (73)	8 (27)		36 (80)	3 (10)	
Correct guess^d^						
Correct	18 (60)	18 (60)		43 (96)	28 (93)	
Incorrect	12 (40)	12 (40)		2 (4)	2 (7)	

a n=45 ChatGPT annotations written by 3 versions of ChatGPT: 3.5, 4, and 5 (ChatGPT, OpenAI) [13]; n=30 human annotations from 2 annotators.

b *f *(%): frequency of response (percent).

c OR: odds ratios estimated from cumulative (for main points and errors) or binary (for quality and context) mixed-effects logit models. Assessor 2’s main points were collapsed into 3 categories (1‐3, 4‐5, and 6‐7) to satisfy the proportional odds assumption. A random intercept for publication was used in all models except for Assessor 2’s evaluation of quality and context, where a single-level fixed-effect model was used. A random intercept for annotators was used in Assessor 1’s evaluations of main points, and Assessor 2’s evaluation of errors. The estimated variances of the random intercepts are reported in Table S8 in the Multimedia Appendix 1.

d Assessor 1 used ChatGPT-5 to produce annotations, so his percentage of correct guesses were out of 30 annotations created by Chat GPT-3.5 and 4 only.

### Main Points, Errors, Quality and Context, and Guesses

We did not find a statistically significant difference in humans’ or ChatGPTs’ odds of presenting a better summary of main points (see Table 2). Although not statistically significant for assessor 1, error ratings were more similar, where both assessors found that humans had lower odds of including errors compared to ChatGPT (Assessor 1: OR 0.31, 95% CI 0.09‐1.02; Assessor 2: OR 0.10, 95% CI 0.03‐0.33). Findings for presence of quality and context were also similar, with both assessors finding strong evidence that humans had lower odds of describing a paper’s quality and context (Assessor 1: OR 0.11, 95% CI 0.03‐0.33; Assessor 2: OR 0.03, 95% CI 0.01‐0.10).

Finding that ChatGPT had higher odds of reporting quality and context is noteworthy given that ChatGPT was not explicitly prompted to do so. However, ChatGPT’s discussion of quality and context was not always appropriate. For instance, in 40% (6/15) of ChatGPT-5’s annotations, it said that these studies may be at risk of “selection bias” because the studies used non-systematic (eg, narrative) review methods to search for eligible studies [17,21-23,25,26]. ChatGPT-5 was correct to point out the limitations of narrative review methods, but did not understand that the correct term in this context was publication bias rather than selection bias.

Furthermore, both assessors found ChatGPT capable of serious errors beyond the use of inaccurate terminology. For example, ChatGPT-5 hallucinated and gave an annotation for a publication on human papillomavirus instead of the assigned publication on diagnostic criteria for temporomandibular disorders [26]. We describe this further and provide additional examples in Table S3 in the Multimedia Appendix 1.

## Discussion

### Principal Findings

Our goal for this exploratory study was to compare the strengths and weaknesses of ChatGPT- and human-generated annotated bibliographies. We did not find a difference in capture of main points across ChatGPT and human annotation. However, we found that ChatGPT-generated summaries had increased odds of being in a worse error category than human summaries. And even though ChatGPT had increased odds of describing a publication’s quality and context, the discussion of the quality and context was sometimes inaccurate.

### Comparison to Prior Work

There are many apps and platforms that build upon existing LLM frameworks (eg, ChatGPT, Claude 3, Gemini, Bard, Co-Pilot, etc) that use more specific training sets to help with specialized tasks [41]. While we do not include a formal evaluation of these more customized GPTs in this study, we expect these approaches would perform better than the base ChatGPT interface on OpenAI’s website because those models were trained on the more specialized scientific literature. Other studies have found that custom GPTs can perform some tasks better than base GPT and this is likely generalizable to other base LLMs and customized models [13,42]. Some have been trained on scientific articles and have access to PubMed and other bibliographic databases—a feature that base ChatGPT does not have—which gives them a greater ability to provide useful contextualization and even suggest potentially related references [12,43,44].

This potential advantages of AI summaries was supported by our finding that chatbot annotations more often provided quality assessment and contextualization than did the human annotations. While the first chatbot prompts did not include a request to contextualize the findings within the broader literature, it may be that the mechanism of an LLM allows it to infer what is “expected” given the request to make a summary for an annotated bibliography. This difference in effective contextualization likely arose because LLMs are predictive models─their very mechanism involves calculation of words in context─thus they may be able to produce an overall summary and extrapolation/contextualization about the literature on a topic based on whatever is available in its training set. Given their access to and training on the scope of their “reading,” this task is relatively simple. However, such a task is very difficult for humans unless they are familiar with the literature already. That said, we found that contextualization was not always appropriate or accurate, with AI summaries sometimes including statements that were not relevant or were incorrect.

### Strengths and Limitations

Our pragmatic approach to this study carries with it the standard strengths and limitations of a naturalistic and exploratory study, including strengths and limitations related to how humans interact with AI. First, one limitation of using chatbots that was reflected in our findings is the varying nature of output and how this was related to the naturalistic prompts that were used. We purposely did not use a standard set of prompts for each version of ChatGPT. Our human annotators were given the same training materials, but they had different levels of experience (by design) and also applied the training differently in generating their summaries. Similarly, in having a different human write their prompt for each chatbot, we had greater variation in the prompt styles that is more generalizable to the real world, in which people are more apt to use their own prompts before finding “standardized” prompts that may have been developed on a different set of literature. This variation is worth noting—while the capacity may be present for AI to produce accurate summaries that are comparable to those produced by humans, people with less experience writing prompts or with barriers such as language fluency might also produce AI-generated summaries that are less effective [45,46].

Further, prompt engineering continues to advance as a technique for producing higher-quality outputs. For example, there are now more advanced ways to prompt which can produce better responses (eg, encoding a prompt a certain way in a JSON file format or directly interfacing with the backend through application programming interfaces. We did not explore these other options or make inferences about prompt engineering techniques, however, because such interaction is likely beyond what the average user is capable of or would undergo for normal use [47].

Second, the interactive and chat-based interface of most LLMs means that the settings within a standard GPT are easily customizable, which may lead to responses being different for each user [48]. Although LLMs are functionally large predictive models with trillions of parameters, given the same prompt, a model will return different results each time a prompt is run [48]. This is a limitation of using these models to summarize literature because different annotations will be produced for the same article. While this variability is also true of multiple human annotators, and both humans and AI can make mistakes, humans may be better able to adapt prompts or instructions to different settings and situations in a way that LLMs cannot. The potential risk is that a carefully written prompt that produces an accurate summary of a paper in one LLM may not produce a reliable or valid summary (1) of a paper that has a different structure (eg, social science vs medical literature) or (2) in an LLM built upon a different framework. Multiple evaluations of LLMs have found the ability to summarize literature accurately varies depending on the context and can be prone to errors such as overgeneralizing statements beyond a given setting [10,49].

We asked both our AI and human annotators for a very specific kind of summary (ie, an annotated bibliography) which is different from other types of summarizations (eg, extractive summaries, descriptive summaries, evaluative summaries, synoptic summaries, etc). This specificity may limit the generalizability of our results to other types of summaries. Another limitation is the potentially low readability of the annotations produced here by both humans and chatbots; however, in our study this may have come from the fact that we were summarizing scientific articles and did not prompt the AI (or instruct the human annotators) to write for a better readability score. Finally, for our original sets of annotations in ChatGPT-3.5 and ChatGPT-4 we fed only article text directly into the LLM: this is a limitation as papers contain multimodal information such as tables and figures which humans may use to make their summaries but may be inaccessible or incomprehensible to LLMs. While this was a potential limitation of the early LLMs that could not handle such information, current versions of LLMs are able to process entire papers including information contained in tables and figures, so we believe the utility should not be affected by this going forward [50].

As our intention was to explore the comparability of AI and human annotations, our sample was small, and we did not power our assessment for any quantitative hypothesis testing. For our generation of annotations, assessments, and comparisons, we followed a predefined protocol as closely as possible.

### Future Directions

Contrary to other studies that look only at AI summaries or answers to questions [5,6,9,51], our findings suggest that LLMs produce summaries of academic articles that are comparable to human annotators in errors and more often include quality assessment and contextualization. While the annotations were not perfect and sometimes needed further prompting to produce better summaries, summaries did not take 10 minutes to create (as they did for human annotations once papers were read) and did not require the experience and training of human researchers. The utility of being able to create summaries without the requisite content expertise at a much lower cost (time * expertise) cannot be understated. Future evaluations should use larger sample sizes that are powered for statistical hypothesis testing instead of exploration and description. It would also be useful for future research to explore AI generation of different kinds of summaries, including single sentence approaches, to deepen our understanding of the use and limitations in different contexts [52]. Future studies should also evaluate the ability of AI to group the studies thematically as this kind of presynthesis would also be helpful for managing libraries of studies in grouping similar concepts together.

### Conclusions

While our findings show promising speed and accuracy of LLMs for producing summaries of papers, when compared with human annotators there is still a risk of errors which necessitates human verification. When approached with these things in mind, we believe the utility is clear, and that human-assisted AI annotation—even through casual and naturalistic interactions with chatbots where output accuracy is verified by a human—will be the most efficient way to create annotated bibliographies that can be used to provide a rapid overview of a breadth of literature for a wide range of users.

## Supplementary material

10.2196/69707 Multimedia Appendix 1 Additional information for Methods and Results.

## Abbreviations

AI: artificial intelligence
FRE: Flesch Reading Ease
LLM: large language model
OSF: Open Science Framework
PICO: population, intervention, comparator, outcome
