# Supplementary material for: Using Large Language Models to Summarize Evidence in Biomedical Articles: Exploratory Comparison Between AI- and Human-Annotated Bibliographies
Source: JMIR Form Res. 2026 Feb 12;10:e69707. doi: 10.2196/69707 (PMC12900274; doi:10.2196/69707)
Supplement: Multimedia Appendix 1 [file formative-v10-e69707-s001.docx]

**Multimedia Appendix**

Table of Contents

[Tables 2](#_Toc213399222)

[Table S1. Description and links to files on Open Science Framework (OSF)^1^ 2](#_Toc213399223)

[Table S2. Examples of scrubbing for annotations 3](#_Toc213399224)

[Table S3. Examples of errors identified in annotations 5](#_Toc213399225)

[Table S4. Contingency table comparing expert raters’ assessment for main points 10](#_Toc213399226)

[Table S5. Contingency table comparing expert raters’ assessment for errors 10](#_Toc213399227)

[Table S6. Contingency table comparing expert raters’ assessment for quality and context 10](#_Toc213399228)

[Table S7. ICC for initial models 11](#_Toc213399229)

[Table S8. Estimates of random-effects variance components 11](#_Toc213399230)

[Boxes 12](#_Toc213399231)

[Box S1. Directions given to human annotators 12](#_Toc213399232)

[Box S2. Directions given to chatbot prompters 12](#_Toc213399233)

[Text S1. Expanded methods and results 13](#_Toc213399234)

[Methods for assessing inter-rater agreement and disagreement 13](#_Toc213399235)

[Methods for modeling assessor-stratified ratings of main points, errors, and quality and context 13](#_Toc213399236)

[Results of assessing inter-rater agreement and disagreement 13](#_Toc213399237)

[Results of modeling of main points, errors, quality and context, and guesses 14](#_Toc213399238)

[Figures 15](#_Toc213399239)

[Figure S1. Agreement of main points evaluation by assessor, publication, and annotator 15](#_Toc213399240)

[Figure S2. Error evaluation by assessor stratified by assessor, publication, and annotator 16](#_Toc213399241)

[Figure S3. Quality and context evaluation by assessor, publication, and annotators 17](#_Toc213399242)

[References 18](#_Toc213399243)

# Tables

## Table S1. Description and links to files on Open Science Framework (OSF)^1^

| File name | Description | Link to file |
| --- | --- | --- |
| Article selection | Literature search described under “Article selection” with resulting articles used in summaries | <https://osf.io/cmt7r/files/vbd89> |
| ChatGPT 3.5 conversations | Prompt used and responses for each article. | <https://osf.io/cmt7r/files/x764v> |
| ChatGPT 4 conversations | Prompt used and responses for each article. | <https://osf.io/cmt7r/files/sud2t> |
| ChatGPT 5 conversations | Prompt used and responses for each article. | <https://osf.io/cmt7r/files/ayh28> |
| Annotation grading_Assessor1 | Raw data file (Assessor 1’s data collection) | <https://osf.io/np7a6> |
| Annotation grading_Assessor2 | Raw data file (Assessor 2’s data collection) | <https://osf.io/pnx2y> |
| annotation.long.2025.10.02.csv | Analysis database | <https://osf.io/cmt7r/files/7fzn2> |
| statistical_code_20251101 | Analysis code (R) | <https://osf.io/cmt7r/files/gtrfb> |

## Table S2**.** Examples of scrubbing for annotations

| GPT version | Selected original text | Scrubbed version used in analysis |
| --- | --- | --- |
| ChatGPT-3.5 | Title: Theta Band Activities and Cognitive Control: A Mechanistic Perspective This article provides an insightful overview of recent advancements in cognitive neuroscience… | This article provides an insightful overview of recent advancements in cognitive neuroscience… |
|  | Annotation: **Annotated Bibliography: Discovery of 2019-nCoV in Wuhan, China** This annotated bibliography summarizes a research report detailing the identification and characterization of a novel coronavirus, 2019-nCoV, associated with a pneumonia outbreak in Wuhan, China, in December 2019. … | This annotated bibliography summarizes a research report detailing the identification and characterization of a novel coronavirus, 2019-nCoV, associated with a pneumonia outbreak in Wuhan, China, in December 2019. … |
|  | Title: Diagnostics for Assessing Balance in Inverse Probability Weighting Using Propensity Scores: A Methodological Review This comprehensive review by Cole and Hernán outlines essential balance diagnostics for assessing the adequacy of assumptions in the context of Inverse Probability of Treatment Weighting (IPTW) using propensity scores. … (Word count: 145) | This comprehensive review by Cole and Hernán outlines essential balance diagnostics for assessing the adequacy of assumptions in the context of Inverse Probability of Treatment Weighting (IPTW) using propensity scores. |
| ChatGPT-4 | Citation (APA): [Author(s)]. ([Year]). [Title of the article]. [Journal Name], [Volume(Issue)], [Page range]. [DOI] Introduction, Aims & Research methods, Scope, Usefulness, Limitations, Conclusions: This study evaluates the National Early Warning Score (NEWS)… | This study evaluates the National Early Warning Score (NEWS)… |
| ChatGPT-4 | Citation (APA): Moons, K. G. M., Altman, D. G., Reitsma, J. B., et al. (2015). Transparent Reporting of a multivariable prediction model for Individual Prognosis Or Diagnosis (TRIPOD): Explanation and Elaboration. Annals of Internal Medicine, 162(1), 55-63. Annotation (70-125 words): This article presents the TRIPOD guideline… | This article presents the TRIPOD guideline, … |
|  | Citation: (Authors not provided, Year). Title not available. Source not available. Introduction: This extensive research study develops and evaluates new WHO cardiovascular disease risk prediction models across 21 global regions… | This extensive research study develops and evaluates new WHO cardiovascular disease risk prediction models across 21 global regions… |

|  |  |  |
| --- | --- | --- |

## Table S3. Examples of errors identified in annotations

| **Annotation version** | **Article title** | **Selected original text** | **Score and rationale** |
| --- | --- | --- | --- |
| ChatGPT-3.5 | The ability of the National Early Warning Score (NEWS) to discriminate patients at risk of early cardiac arrest, unanticipated intensive care unit admission, and death^2^ | *“This comprehensive study explores the effectiveness of the National Early Warning Score (NEWS) in predicting adverse outcomes among patients admitted for acute medical care. The research evaluates NEWS against 33 other early warning scoring systems and uses a large vital signs database from over 35,000 patient episodes. The results demonstrate that NEWS performs well as a discriminator of patients at risk of cardiac arrest, unanticipated intensive care unit (ICU) admission, or death within 24 hours. Compared to other systems, NEWS exhibits superior ability to identify patients at risk of combined outcomes. However, its predictive ability for cardiac arrest alone is less pronounced.* ***While this study supports the standardization of NEWS in clinical practice****, it also emphasizes the need for ongoing refinement and evaluation of early warning scoring systems.”* | **Minor error**:  The authors do not indicate that the tool should be standard in clinical practice but acknowledge that this was a single-center study that requires external validation and it may not work if implemented elsewhere. |
|  | The Fear of COVID-19 Scale: Development and Initial Validation^3^ | *“In this research article, the authors present the development and validation of the Fear of COVID-19 Scale (FCV-19S).* ***The scale, comprising seven items, aims to assess individuals' fears related to the COVID-19 pandemic.*** *The study involved 717 Iranian participants, and the psychometric properties of the scale were thoroughly evaluated using classical test theory and Rasch model analysis. The results indicated that the FCV-19S exhibits robust psychometric properties, with satisfactory internal consistency, reliability, and concurrent validity.* ***The scale's unidimensional structure allows for easy and reliable assessment of fear related to COVID-19 among individuals of various genders and ages.*** *The FCV-19S appears to be a valuable tool for understanding and addressing psychological issues arising from the pandemic.”* | **Multiple errors**:  The scale was meant to assess fears of COVID-19 virus and not the COVID-19 pandemic. Stating that measurement a unidimensional structure makes it easy to assess in different gender and age groups is incorrect, this concept refers to differential item functioning and whether measurement invariance is present. |
| ChatGPT-4 | The REDCap consortium: Building an international community of software platform partners^4^ | *“This article documents the* ***global expansion of the REDCap Consortium between 2013-2018****. It aims to explore the software development and dissemination model utilized by REDCap, detailing its growth, functionality, challenges, and benefits.* ***The research employs a qualitative analysis*** *of growth patterns, integration strategies, and development philosophies. While showcasing the success of the REDCap model in different environments****, it also highlights some restrictions, such as the limitation to non-profit organizations****. The article concludes by underscoring the Consortium's effective model for software development and sharing, offering insights for similar projects.”* | **Multiple errors**:  The expansion from Vanderbilt began as early as 2005; the paper is descriptive with no “analysis”; the consortium includes academic and government organizations in addition to non-profits. |
|  | The Fear of COVID-19 Scale: Development and Initial Validation^3^ | “***This research conducted by Wang et al. (2020****) addresses the emergent issue of the psychological impact of the COVID-19 pandemic, specifically fear and its manifestations. The authors utilized psychometric testing and Rasch analysis to develop and validate the Fear of COVID-19 Scale (FCV-19S), demonstrating the urgency of acknowledging and addressing the psychological implications of this health crisis. The study's focus extends beyond physical health and infection control, shedding light on an overlooked aspect of pandemic management. However, it's limited to the Iranian population and its methodological approach restricts generalizability.* ***The FCV-19S, nevertheless, serves as a crucial instrument to measure and manage fear, aiding healthcare providers in designing appropriate mental health interventions****.*” | **Multiple errors**:  A paper cited in the background section (Wang et al. 2020) was indicated to be the author of the study. The tool was not constructed with the intent to specifically design mental health interventions. |
| ChatGPT-5 | Diagnostic Criteria for Temporomandibular Disorders (DC/TMD) for Clinical and Research Applications: recommendations of the International RDC/TMD Consortium Network* and Orofacial Pain Special Interest Group^5^ | *“****This review outlines the natural history of human papillomavirus (HPV) infection and its role in the multistage development of cervical cancer, moving from initial infection to persistence, progression to precancerous lesions, and eventual invasive carcinoma****. The authors highlight the centrality of high-risk HPV types, viral persistence, and host–viral interactions, while also examining the influence of cofactors such as immune status and smoking. The paper is valuable for its clear synthesis of HPV-driven carcinogenesis and its implications for screening and vaccination strategies, though as a narrative review it may introduce selection bias and underrepresent less common or conflicting evidence. Its effectiveness lies in bridging epidemiology with molecular mechanisms, offering clinicians and researchers a concise framework for understanding cervical cancer prevention and control.”* | **Major error**:  The AI generated a summary of a completely different paper than the .pdf that was given to it. |
|  | Transparent reporting of a multivariable prediction model for individual prognosis or diagnosis (TRIPOD): the TRIPOD statement^3^ | “*This article introduces the TRIPOD Statement, a 22-item checklist developed to improve transparency and completeness in reporting studies on diagnostic and prognostic prediction models.* ***The statement was created through systematic review****, expert survey, and consensus meetings, emphasizing the need for clear descriptions of study design, predictors, outcomes, validation, and performance measures. It is a valuable guideline that enhances reproducibility, critical appraisal, and potential implementation of prediction models in clinical practice, though it does not prescribe methods or serve as a quality assessment tool. A limitation is that adherence depends on researchers and journals, leaving the risk that methodological weaknesses could still persist despite improved reporting.*” | **Major error**:  TRIPOD was not developed from a systematic review, which is a specific study design. Instead, the authors conducted a systematic search of the literature, but they did not conduct a systematic review. |
| Human 2 | A Novel Coronavirus from Patients with Pneumonia in China, 2019^6^ | *“This study uses lower respiratory tract samples from three patients hospitalized in Wuhan, China in December 2019* ***and January 2020*** *to isolate and identify a novel coronavirus 2019-nCoV that caused pneumonia in patients. The study used whole-genome sequencing, directed PCR, and culture. The authors conclude that this virus is the likely causative agent of the viral pneumonia outbreak in Wuhan.”* | **Major error**:  The patients were hospitalized and samples collected in December of 2019. |
|  | Moving towards best practice when using inverse probability of treatment weighting (IPTW) using the propensity score to estimate causal treatment effects in observational studies^7^ | “***This article is a systematic review*** *of a statistical technique called inverse probability of treatment weighting (IPTW) that uses propensity scores to generate synthetic samples in which treatment assignment is independent of measured baseline covariates. This methodology can be used for estimating treatment effects from observational data when random assignment of participants is not possible. The paper reviews methods of IPTW, the literature that has used IPTW, and model diagnostics. It also presents a case study illustrating the application of IPTW, and finally provides a discussion and recommendations for practice. The authors conclude that while these methods have been increasingly used in the scientific literature, many recent studies did not examine whether weighting balanced covariates between treatment groups. They provide described diagnostics for assessing the balance of baseline covariates between treatment groups in the hope that increased use of these diagnostics will improve the practice of IPTW for estimating average treatment effects using observational data.*” | **Major error**:  The study design of the paper was not a systematic review. |

## Table S4. Contingency table comparing expert raters’ assessment for main points

| **Assessor 1** | **Assessor 2** | | | | | | |
| --- | --- | --- | --- | --- | --- | --- | --- |
|  | **1** | **2** | **3** | **4** | **5** | **6** | **7** |
| **1** | 1 | 0 | 0 | 0 | 0 | 0 | 0 |
| **2** | 0 | 0 | 0 | 0 | 0 | 0 | 0 |
| **3** | 0 | 0 | 0 | 2 | 3 | 0 | 0 |
| **4** | 2 | 0 | 0 | 4 | 1 | 2 | 0 |
| **5** | 1 | 1 | 0 | 2 | 7 | 1 | 0 |
| **6** | 2 | 1 | 1 | 4 | 3 | 13 | 1 |
| **7** | 0 | 2 | 1 | 0 | 4 | 13 | 3 |

## Table S5. Contingency table comparing expert raters’ assessment for errors

| **Assessor 1** | **Assessor 2** | | | |  |
| --- | --- | --- | --- | --- | --- |
|  | **1 (None)** | **2 (One minor)** | **3 (One major)** | **4 (Multiple)** | |
| **1** | 34 | 6 | 3 | 11 | |
| **2** | 3 | 3 | 2 | 2 | |
| **3** | 1 | 0 | 1 | 0 | |
| **4** | 0 | 0 | 0 | 9 | |

## Table S6. Contingency table comparing expert raters’ assessment for quality and context

| **Assessor 1** | **Assessor 2** | |
| --- | --- | --- |
|  | **No** | **Yes** |
| **No** | 25 | 9 |
| **Yes** | 11 | 30 |

## Table S7. ICC for initial models

| **Model** | **Publication ICC** | **Annotation source ICC** |
| --- | --- | --- |
| Assessor 1’s evaluation of main points | 0.264 | 0.201 |
| Assessor 2’s evaluation of main points | 0.078 | 0.004 |
| Assessor 1’s evaluation of errors | 0.170 | 0.001 |
| Assessor 2’s evaluation of errors | 0.258 | 0.180 |
| Assessor 1’s evaluation of quality and context | 0.040 | 0.471 |
| Assessor 2’s evaluation of quality and context | NA | NA |

## Table S8. Estimates of random-effects variance components

| **Model** | **Variance- Publication** | **Variance-**  **Annotation source** |
| --- | --- | --- |
| Assessor 1’s evaluation of main points | 1.273 | 1.110 |
| Assessor 2’s evaluation of main points | 0.480 | Not used |
| Assessor 1’s evaluation of errors | 0.901 | Not used |
| Assessor 2’s evaluation of errors | 1.321 | <0.001 |
| Assessor 1’s evaluation of quality and context | 0.513 | Not used |
| Assessor 2’s evaluation of quality and context | Not used | Not used |

# Boxes

## Box S1**.** Directions given to human annotators

| Please write critical/evaluative annotations for the following open access articles. The full text of each article is linked below the citation.  You can use these resources to learn more about annotated bibliographies:   - How to Write an Annotated Bibliography (https://www.youtube.com/watch?v=Il_1Q3HZLhA) - The Writing Center-Annotated Bibliographies (https://writingcenter.unc.edu/tips-and-tools/annotated-bibliographies/) - Assessing and presenting summaries of evidence in Cochrane Reviews (https://systematicreviewsjournal.biomedcentral.com/articles/10.1186/2046-4053-2-81)   Please keep each annotation to one paragraph and note how long it takes you to complete each annotation. Please return all annotations within one week. Please contact MCC ASAP if you have any questions or run into any problems. |
| --- |

## Box S2. Directions given to chatbot prompters

| 1. Use [https://chatgpt-prompt-splitter.jjdiaz.dev/](https://nam02.safelinks.protection.outlook.com/?url=https%3A%2F%2Fchatgpt-prompt-splitter.jjdiaz.dev%2F&data=05%7C02%7Ckevin.naaman%40cuanschutz.edu%7C57c705100d4f424d80ea08de07e2e388%7C563337caa517421aaae01aa5b414fd7f%7C0%7C0%7C638956868263197594%7CUnknown%7CTWFpbGZsb3d8eyJFbXB0eU1hcGkiOnRydWUsIlYiOiIwLjAuMDAwMCIsIlAiOiJXaW4zMiIsIkFOIjoiTWFpbCIsIldUIjoyfQ%3D%3D%7C0%7C%7C%7C&sdata=O%2BTwm8Bg0RchdHGRM%2Brscce%2FTAfmEkaFcMRtaMjczsk%3D&reserved=0) to split an article into parts. 2. The splitter will tell ChatGPT to wait for all of the pieces, then to be ready to respond to requests. 3. Copy and paste each part using the buttons on the website 4. When it acknowledges that it has received ALL parts, use this prompt:    1. “Please summarize that text in an evaluative annotated bibliography format of between 90 and 150 words.” 5. Copy and paste the resulting summary into the annotated bibliography. Do not ask it to regenerate the response or do anything else, even if it’s completely wrong (but do let me know if a few of them are completely wrong so we can tweak as necessary). 6. Start a new conversation for the next article and follow the same steps 7. Download each conversation into a single Google Doc 8. Share the Google doc with the annotations and the conversations. |
| --- |

***Note.*** *The link* [*https://chatgpt-prompt-splitter.jjdiaz.dev/*](https://nam02.safelinks.protection.outlook.com/?url=https%3A%2F%2Fchatgpt-prompt-splitter.jjdiaz.dev%2F&data=05%7C02%7Ckevin.naaman%40cuanschutz.edu%7C57c705100d4f424d80ea08de07e2e388%7C563337caa517421aaae01aa5b414fd7f%7C0%7C0%7C638956868263197594%7CUnknown%7CTWFpbGZsb3d8eyJFbXB0eU1hcGkiOnRydWUsIlYiOiIwLjAuMDAwMCIsIlAiOiJXaW4zMiIsIkFOIjoiTWFpbCIsIldUIjoyfQ%3D%3D%7C0%7C%7C%7C&sdata=O%2BTwm8Bg0RchdHGRM%2Brscce%2FTAfmEkaFcMRtaMjczsk%3D&reserved=0) *now has a security error but the code can be found at* [*https://github.com/jupediaz/chatgpt-prompt-splitter*](https://github.com/jupediaz/chatgpt-prompt-splitter)*.*

# Text S1. Expanded methods and results

## Methods for assessing inter-rater agreement and disagreement

To assess agreement and disagreement between the two assessors, we applied Cohen’s 𝜅 for the quality and context binary rating and weighted Cohen’s 𝜅 for the ordinal variables (main points and errors). Following the recommendations by Agresti,^8^ we also investigated patterns for systematic disagreement by applying McNemar’s $\chi^{2}$ for the quality and context rating (without continuity correction) and ordinal quasi-symmetry models for main points and errors.

## Methods for modeling assessor-stratified ratings of main points, errors, and quality and context

We fit a binary logit mixed-effects model with quality and context as the outcome and separate cumulative logit mixed-effects models with main points and errors as the outcomes. Our goal was to fit assessor-stratified models with a binary indicator for annotation source (human versus ChatGPT) and random intercepts to account for clustering (1) at the publication level and (2) for the five annotators.

Before fitting final models, we first determined whether random intercepts for the 15 publications and the 5 annotators were needed by evaluating the magnitude of the intraclass correlation coefficients (ICCs) from the models fitted with only the random intercepts (i.e., the empty models). If the ICCs for any of the random intercepts were near 0, we determined that clustering was negligible and dropped the random intercept(s) from the model(s). For the cumulative logit models, we assessed the proportional odds assumption using the nominal_test function from the *ordinal* package,^9^ and if violated, we collapsed neighboring categories and retested the proportional odds assumption. For transparency, we report the ICCs, results from the nominal test, and our final modeling decisions in the results section below.

Statistical significance for outcomes modeling was defined as p<0.05. However, because the goal of our analysis was exploratory, we reported our results with a focus on describing our observed data rather than intending to draw inferences to a population of studies beyond the scope of this paper.

## Results of assessing inter-rater agreement and disagreement

Raw scoring by assessor and results of stratified modeling are reported in the Results section of the main paper, while contingency tables comparing assessor’s scoring (TablesS4-S6), formal assessments of agreement with Cohen’s $\kappa$, and patterns of disagreement with McNemar’s $\chi^{2}$ and ordinal quasi-symmetry models are reported below.

Weighted Cohen’s 𝜅 was 0.32 for main points (95% CI 0.11-0.52; raw agreement = 37%, n = 28/75). Although the estimate for agreement between assessors was fair, ordinal quasi-symmetry models revealed systematic disagreement where one assessor was consistently stricter than the other assessor. For main points, the odds that the stricter assessor would assign a higher rating were approximately 51% lower per category step (OR = 0.49, 95% CI 0.30-0.71, *p* = 0.001) compared to the other assessor.

Weighted Cohen’s 𝜅 was 0.43 for errors (95% CI 0.23-0.63; raw agreement = 63%, n = 47/75). Although the estimate for agreement between assessors was moderate, ordinal quasi-symmetry models revealed systematic disagreement where one assessor was consistently stricter than the other assessor. For errors, the odds of assigning a worse category per step were approximately three times greater (OR = 3.05, 95% CI 1.69-7.56, *p =* 0.002) than the other assessor.

Cohen’s 𝜅 for quality and context was 0.46 (95% CI 0.26-0.67; raw agreement = 73%, n = 55/75), and there was no evidence of systematic disagreement (McNemar’s *2*(1) = 0.2, *p* = 0.655).

## Results of modeling of main points, errors, quality and context, and guesses

We began our analyses by first assessing the proportional odds assumption of the ordinal variables. All models met the assumption except for Assessor 2’s evaluation of main points, where the proportional odds assumption was later satisfied after collapsing main points into three categories (1-3, 4-5, and 6-7). Intraclass correlation coefficients for the models where we included both random intercepts without any predictor variables are reported in Table S7.

Cells are highlighted in blue if a random intercept was needed to account for clustering at the level of publication (15 publications in total) or level of annotation source (5 sources in total). Red cells indicate a random intercept was not needed due to small values of ICC. Due to issues with singularity for Assessor 2’s evaluation of quality and context, we fit a single-level, fixed-effect logit model.

Resultant estimates of the random-effects variance components are reported below. We did not fit a random intercept for Assessor 1’s evaluation of quality and context as planned because the fixed effects were not numerically consistent. We therefore only modeled the random intercept for publication.

# Figures

## Figure S1. Agreement of main points evaluation by assessor, publication, and annotator


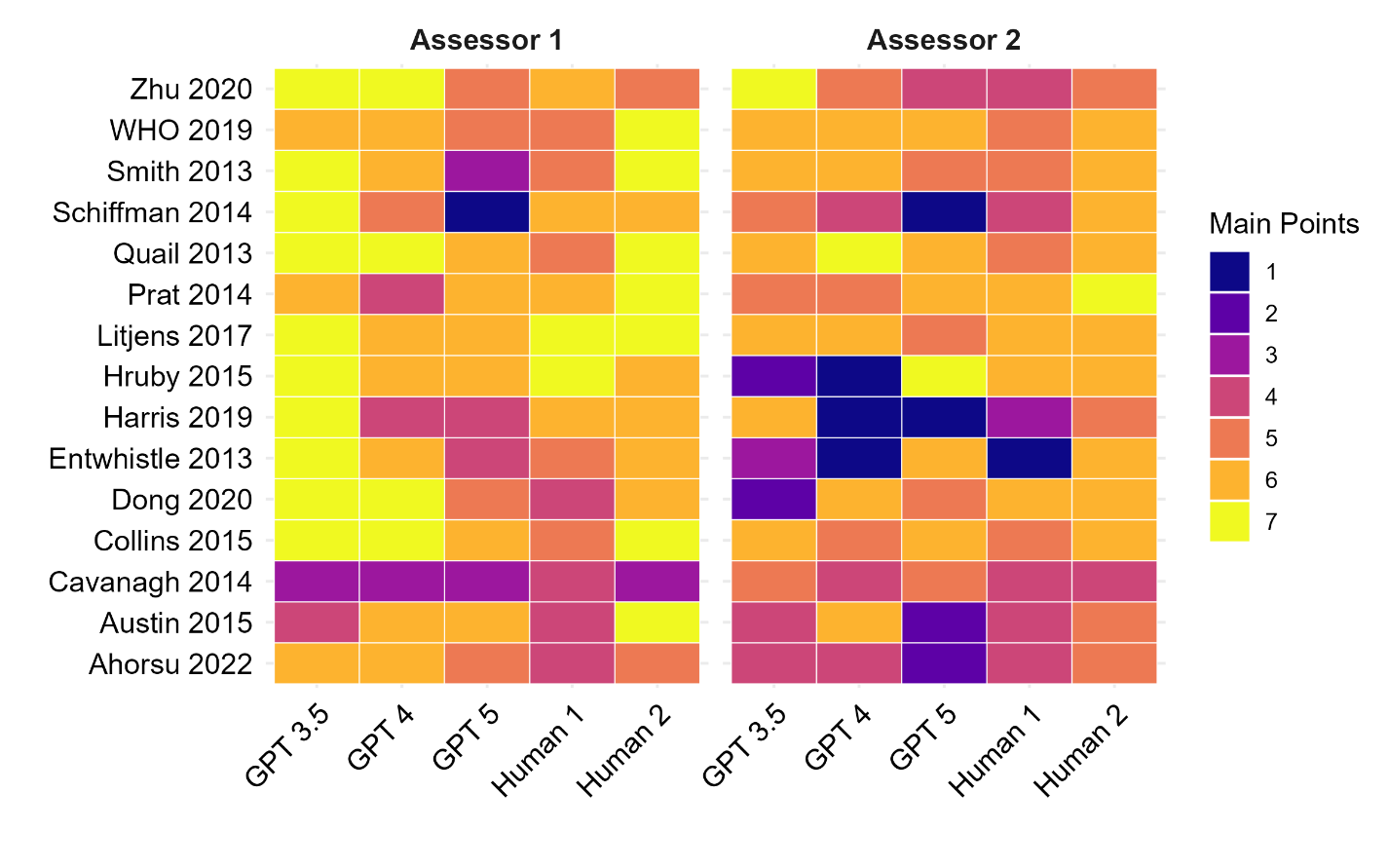


## Figure S2. Error evaluation by assessor stratified by assessor, publication, and annotator


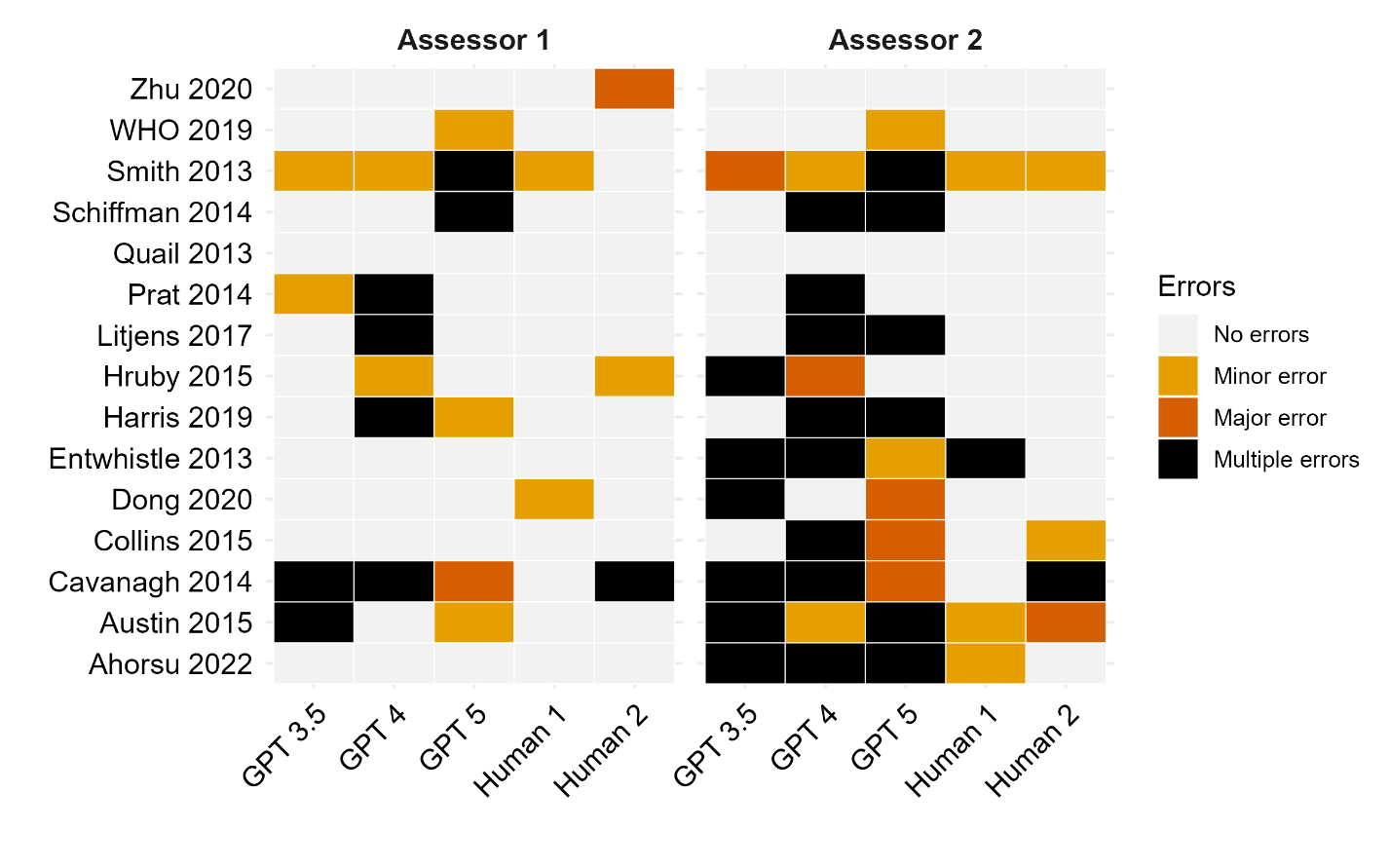


## Figure S3. Quality and context evaluation by assessor, publication, and annotators


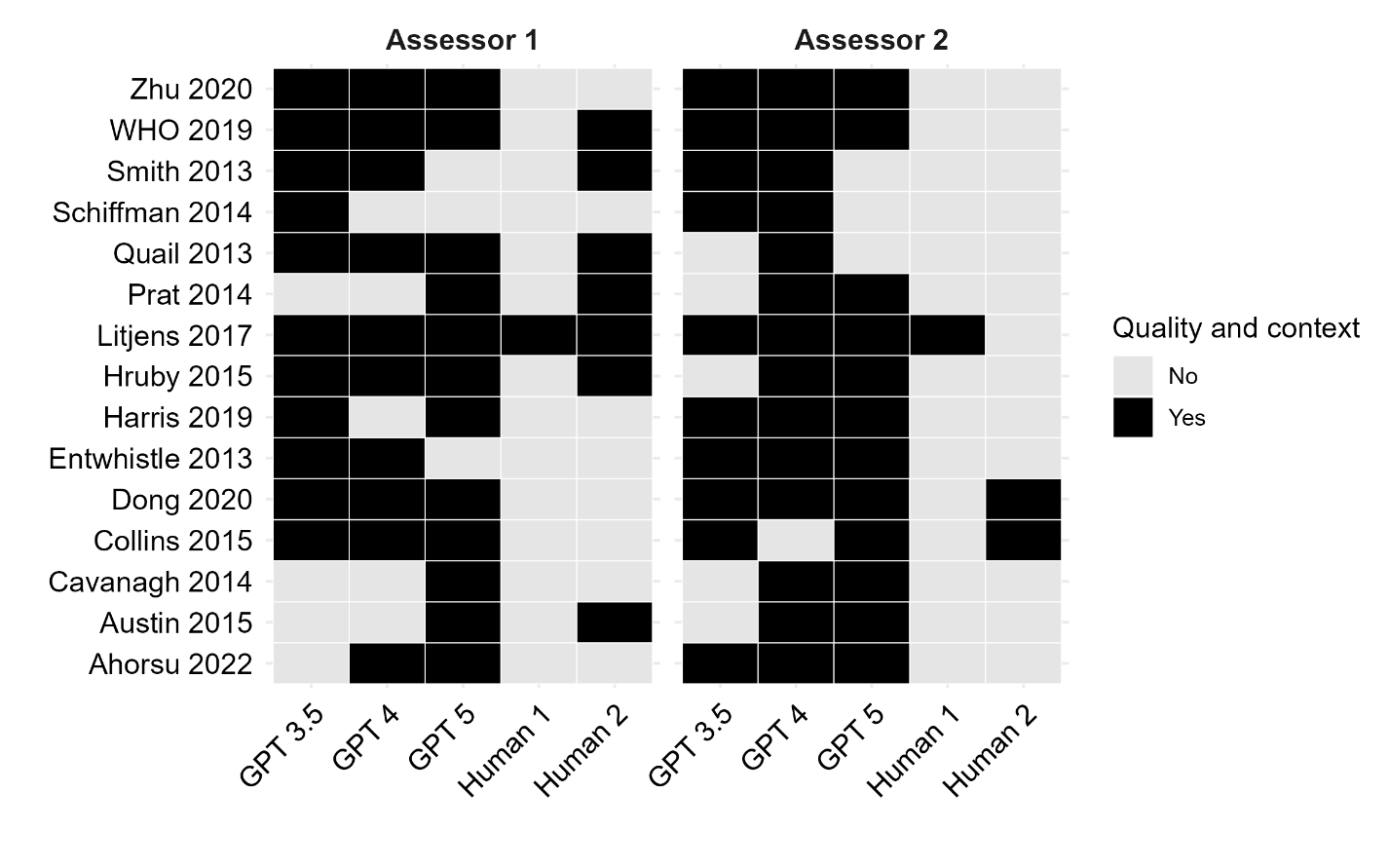


# References

1. AI Generation of Evidence Summaries. OSF.io. March 27, 2024. Accessed November 4, 2025. https://osf.io/cmt7r

2. Smith GB, Prytherch DR, Meredith P, Schmidt PE, Featherstone PI. The ability of the National Early Warning Score (NEWS) to discriminate patients at risk of early cardiac arrest, unanticipated intensive care unit admission, and death. *Resuscitation*. 2013;84(4):465-470. doi:10.1016/j.resuscitation.2012.12.016

3. Ahorsu DK, Lin CY, Imani V, Saffari M, Griffiths MD, Pakpour AH. The Fear of COVID-19 Scale: Development and Initial Validation. *Int J Ment Health Addict*. 2022;20(3):1537-1545. doi:10.1007/s11469-020-00270-8

4. Harris PA, Taylor R, Minor BL, et al. The REDCap consortium: Building an international community of software platform partners. *J Biomed Inform*. 2019;95:103208. doi:10.1016/j.jbi.2019.103208

5. Schiffman E, Ohrbach R, Truelove E, et al. Diagnostic Criteria for Temporomandibular Disorders (DC/TMD) for Clinical and Research Applications: recommendations of the International RDC/TMD Consortium Network* and Orofacial Pain Special Interest Group†. *J Oral Facial Pain Headache*. 2014;28(1):6-27. doi:10.11607/jop.1151

6. Zhu N, Zhang D, Wang W, et al. A Novel Coronavirus from Patients with Pneumonia in China, 2019. *N Engl J Med*. 2020;382(8):727-733. doi:10.1056/NEJMoa2001017

7. Austin PC, Stuart EA. Moving towards best practice when using inverse probability of treatment weighting (IPTW) using the propensity score to estimate causal treatment effects in observational studies. *Stat Med*. 2015;34(28):3661-3679. doi:10.1002/sim.6607

8. Agresti A. Modelling patterns of agreement and disagreement. *Stat Methods Med Res*. 1992;1(2):201-218. doi:10.1177/096228029200100205

9. Christensen RHB. ordinal: Regression Models for Ordinal Data. Published online August 19, 2024. Accessed November 4, 2025. https://cran.r-project.org/web/packages/ordinal/index.html
